# Serum haptoglobin concentration and liver enzyme activity as indicators of systemic inflammatory response syndrome and survival of sick calves

**DOI:** 10.1111/jvim.16357

**Published:** 2022-01-18

**Authors:** Camilo Jaramillo, David L. Renaud, Luis G. Arroyo, Daniel G. Kenney, Lisa Gamsjaeger, Diego E. Gomez

**Affiliations:** ^1^ Department of Clinical Studies, Ontario Veterinary College University of Guelph Guelph Ontario Canada; ^2^ Department of Population Medicine, Ontario Veterinary College University of Guelph Guelph Ontario Canada; ^3^ University of Zurich, Vetsuisse Faculty, Bovine Medicine Department Zurich Switzerland; ^4^ Present address: William R. Pritchard Veterinary Medical Teaching Hospital, School of Veterinary Medicine University of California‐Davis Davis California USA

**Keywords:** acute phase proteins, calf, GGT, GLDH, mortality

## Abstract

**Background:**

Increased concentration of haptoglobin (Hp) in serum is associated with survival of critically ill humans and horses. High serum activity of liver‐derived enzyme is associated with sepsis in children and foals.

**Hypothesis/Objectives:**

Investigate whether admission serum Hp and glutamic dehydrogenase (GLDH) are associated with systemic inflammatory response syndrome (SIRS) and survival of sick calves.

**Animals:**

One hundred two calves.

**Methods:**

Retrospective cross‐sectional study. Electronic medical records from all calves <30 days of age admitted to a teaching hospital for 8 years were reviewed. The signalment, clinicopathological findings, the presence of SIRS, final diagnosis, hospitalization time and outcome were recorded. A Cox proportional hazard ratio (HzR) were calculated to assess the association between clinicopathological variables and survival to discharge.

**Results:**

Serum Hp concentrations were similar between SIRS (0.29 g/L; range, 0.05‐3.6) and non‐SIRS calves (0.22 g/L; range, 0‐4.2; *P* = .62). GLDH activity was similar between SIRS (12 U/L; range, 1‐1025) and non‐SIRS calves (9 U/L; range, 2‐137; *P* = .2). Absent suckle reflex (HzR: 6.44, 95% CI: 1.44‐28.86), heart rate (HR) < 100 beats per minute (bpm; HzR: 12.2; 95% CI: 2.54‐58.62), HR > 140 bpm (HzR: 3.59, 95% CI: 1.05‐12.33), neutrophil count <1.7 × 10^9^/L (HzR: 7.36; 95% CI: 2.03‐26.66) and increased gamma‐glutamyl transferase activity (every 50‐unit, HzR: 1.12; 95% CI: 1.03‐1.21) were predictive of nonsurvival.

**Conclusions and Clinical Importance:**

The use of Hp and GLDH for prediction of survival in sick calves cannot be recommended at this time.

**Abbreviations**
ALP: alkaline phosphatase
ALT: alanine aminotransferase
AST: aspartate aminotransferase
FTPI: failure of transfer of passive immunity
GGT: gamma‐glutamyltransferase
GLDH: glutamic dehydrogenase
Hp: haptoglobin
HzR: hazard ratios
SDH: sorbitol dehydrogenase
SIRS: systemic inflammatory response syndrome

## 1 INTRODUCTION

Haptoglobin (Hp) is a positive acute phase inflammatory protein that is produced by the liver, and increased production during inflammation is stimulated by interleukin‐1, interleukin‐6, and tumor necrosis factor α released from macrophages and monocytes at the site of inflammation.^1^, ^2^ The anti‐inflammatory properties of Hp are associated with its antioxidant activity because hemoglobin binding to Hp prevents heme and iron release, which reduce radical oxygen species generation.^3^ Haptoglobin (Hp) improves immune‐tolerance via suppressing the proinflammatory mediators and plays a role in preventing bacterial proliferation during bacteremia.^4^ In humans, increased serum Hp concentrations are associated with decreased in‐hospital mortality because sepsis, whereas an increased risk of mortality occurs in septic subjects with increased concentrations of circulating free‐hemoglobin because of low serum Hp concentrations.^5^ There is increased concentration of Hp in calves with diarrhea,^6^ respiratory disease complex^7^, ^8^ and during experimentally induced *Salmonella* Dublin infection.^9^ In those diseases, serum Hp concentration correlates positively with the severity of illness.^6^, ^9^ In bovine medicine, Hp concentration is utilized in cattle to assist in the diagnosis and prediction of several diseases (eg, pneumonia, traumatic reticuloperitonitis, metritis),^7^, ^8^, ^10^, ^11^, ^12^ however, evidence to suggest that Hp at hospital admission is associated with clinical outcomes of sick calves is lacking. Specifically, it is unknown whether Hp concentration of hospitalized calves is only a marker of severity of disease (eg, systemic inflammatory response syndrome) or if increased Hp concentration during disease have a protective function and are associated with improved survival.

Assessment of hepatic necro‐inflammatory activity and cholestasis in large animals requires the measurement of serum activity of liver‐derived enzyme including serum aspartate aminotransferase (AST), glutamic dehydrogenase activity (GLDH), sorbitol dehydrogenase (SDH), gamma‐glutamyltransferase (GGT), alkaline phosphatase (ALP). In ruminants, GLDH is a sensitive indicator of hepatic necrosis, more specific than AST, and has a better in vitro storage ability than SDH.^13^ In children, high serum activity of liver‐derived enzyme is commonly identified in hospitalized subjects and increased activity is associated with death.^14^ In foals, serum activity of liver‐derived enzyme (GGT and SDH) is increased during sepsis, but their concentrations are not associated with survival.^15^ Calves are frequently presented to tertiary hospitals with primary diseases associated with systemic alterations such as hypoxia, hypoperfusion, or sepsis that can lead to hepatic injury. However, the association between serum activity of liver‐derived enzyme with SIRS or survival during hospitalization of sick calves is yet to be described. The objectives of this study were: (a) To describe serum Hp concentration and GLDH activity and other clinicopathological variables in sick hospitalized calves, particularly in calves suffering from SIRS; and (b) to investigate whether admission serum Hp and GLDH and other clinicopathological variables in hospitalized sick calves are associated with survival to hospital discharge. We hypothesized that admission serum Hp concentration and GLDH activity are associated with SIRS and outcome (survival) of sick calves.

## 2 MATERIALS AND METHODS

### 2.1 Animals

Electronic medical records from all calves <30 days of age admitted to the Ontario Veterinary College of the University of Guelph from January 2011 through December 2019 were reviewed. Calves were included if: (a) their history and physical exam indicated they were admitted because of compromised health status, and (b) serum biochemistry profile including Hp concentrations and GLDH activity were measured upon admission using a serum biochemistry multianalyzer (Cobas 6000 C501; Roche Canada, Laval Quebec, Canada).

Signalment information extracted from each record was: breed, sex (male, female), and age (days). Recorded physical examination findings included attitude (bright, obtunded, or stuporous), posture (standing, sternal or lateral recumbence), suckling reflex (strong, weak, or absent), heart rate (HR, beats/minute, bpm), respiratory rate (RR, respirations/minute, rpm), mucus membranes (normal, congestive, pale, toxic), rectal temperature (*T*, °C), and degree of dehydration estimated based on clinical signs by the attending clinician (5%‐6%, mild dehydration; 7%‐9%, moderate dehydration; >10%, severe dehydration). Clinicopathologic data extracted from the complete blood cell count analysis performed using an Advia 2120i (Sofware 6.9.0—MS, Siemens Healthcare GmbH, Erlangen, Germany) included: Complete white blood cell count (WBC, ×10^9^ cells/L), absolute neutrophils count (×10^9^ cells/L), lymphocytes (×10^9^ cells/L), monocytes (×10^9^ cells/L), basophils (×10^9^ cells/L), eosinophils (×10^9^ cells/L), bands counts (×10^9^ cells/L), PCV (%) and total red blood cell count (×10^12^ cells/L). The following data were extracted from the serum biochemistry profile: total protein (g/L), albumin (g/L), globulin (g/L), Alb/Glob ratio, urea (mmol/L), creatinine (μmol/L), glucose (mmol/L), beta‐hydroxybutyrate (BHBA, μmol/L), haptoglobin (g/L) and GGT (U/L), AST (U/L), GLDH (U/L) and creatine kinase (CK, U/L) activities. The biochemistry analysis was performed using the Cobas 6000 c501 (Roche, Software # 7277112‐05‐01) chemistry analyzer. Creatinine concentration was measured using the Kinetic Jaffe's reaction with alkaline picrate and urea concentration was determined using the Urea Urease‐GLDH kinetic assay. Glucose concentration was measured using the hexokinase/G6PD method, total protein concentration with the biuret method and albumin concentration with the Bromocresol Green colorimetric reaction. The catalytic activity concentrations of GGT, AST, GLDH and CK were determined using enzymatic colorimetric assays. The BHBA concentration was determined spectrophotometrically (optical; Roche. Software # 7277112‐05‐01). Haptoglobin concentrations were measured by determining the hemoglobin binding capacity of serum, which was quantified against a standard sample.^16^ From the blood gas analysis, the blood pH, venous PCO_2_ (PvCO2, mm Hg), HCO_3_^−^ (mmol/L), L‐lactate (L‐Lact, mmol/L) and ionized calcium (iCa, mmol/L) concentrations were recorded. Blood gas analysis was performed using an ABL Flex Plus (Sofware 3.3, Radiometer, Mississauga, Canada) analyzer.

Hospitalization time (days), survival to discharge (yes, no), and the final diagnosis determined by the attending clinician or postmortem examination results or both were also recorded. Calves were considered to have SIRS if they met at least 2 of the following criteria^17^: leukocytosis (WBC count >13.3 × 10^9^ cells/L), leukopenia (WBC count <5.05 × 10^9^ cells/L); hypothermia (*T* < 38.5°C [101.5°F]) or hyperthermia (*T* > 39.4°C [103°F]); tachycardia (HR ≥140 bpm) or bradycardia (HR < 100 bpm); and tachypnea (RR > 60 rpm).

### 2.2 Statistical analysis

Normality of the data was evaluated using the Kolmogorov‐Smirnov test. The mean and SD were calculated for normally distributed variables and the median and ranges were determined for nonnormally distributed variables. Numerical variables were compared between groups (eg, SIRS vs non‐SIRS and survivors vs nonsurvivors) using a *t*‐student or Mann‐Whitney *U*‐tests, depending on the normality of the data, whereas categorical variables were compared using the Fisher exact or *X* ^2^ tests.

A Cox proportional hazard model was constructed to assess the impact of potential predictor variables on the outcome of interest (survival). Days from admission to the hospital until discharged or died/euthanized was used as the time variable. Variables evaluated in the model were sex, breed, attitude, posture, suckling reflex, HR, RR, rectal temperature (*T*), mucus membranes, estimated concentration of dehydration, total WBC, absolute counts of neutrophils, lymphocytes, monocytes, basophils, and eosinophils, serum total protein, albumin, glucose, urea, creatinine, GGT, AST, CK, GLDH, BHBA, and Hp. Martingale residuals were plotted against the continuous variables to assess the linearity of each continuous predictor variable offered to the cox proportional hazard models. If the variable failed to meet the linearity assumption, it was categorized based on previously published reference ranges or references ranges provided by the institutional animal health laboratory (Table S1). Collinearity among variables was tested using Pearson correlation coefficients and if *r* ≥ .6, only 1 variable was retained in the model. Variables with moderate statistical associations (*P* < .2) with the outcome of interest in univariable models were subsequently included in the multivariable analyses. A manual forward stepwise multivariable regression analysis was used for the model. Variables were retained in the multivariable model if *P* < .05 or if the effect of removing the variable resulted in a change of at least 20% in the coefficient of a significant variable, indicating potential confounding. Two‐way interactions were evaluated between variables based on evidence from the literature and retained in the model if significant. Model fit was assessed using the test of proportional hazards for the cox proportional hazards models. The Harrell's C concordance statistic for each survival model was calculated to evaluate the overall predictive ability. *P*‐values < .05 were considered significant. Statistical analyses were performed using Minitab 19 (Minitab, LLC, State College, PA) and STATA 16 (StataCorp LLC, College Station, TX) statistical software.

## 3 RESULTS

A total of 102 of 267 calves met the inclusion criteria, 42 (41%) males and 60 (59%) females. Included breeds were Holstein Friesian (90, 88%), Jersey (7, 7%) and others breeds (5, 5%; 2 Angus, 2 Speckle Park, and 1 mix‐breed). The median age at admission was 12.5 days (range, 1‐30). Seventy‐six (74%) calves had diarrhea, 32 (31%) had pneumonia, 28 (27%) had sepsis and 10 (10%) had gastrointestinal diseases other than diarrhea (ie, mesentery volvulus [n = 2], abomasal ulcers [n = 2], hepatitis [n = 2], constipation [n = 2], ileus [n = 1], inguinal hernia [n = 1]). Concurrent diarrhea and pneumonia were documented in 19 (18%) calves, whereas concurrent diarrhea, pneumonia and sepsis were diagnosed in 10 (10%) patients. The overall hospitalization time was 6 days (range, 1‐24). The proportion of calves that survived was 78% (n = 80).

### 3.1 SIRS and non‐SIRS calves

Eighty‐four (82%) calves had available data for SIRS classification. Forty‐nine (58%) of 84 calves were considered as having SIRS. Calves with SIRS had a significantly lower temperature than non‐SIRS calves; however, the median temperature for both groups was within the normal ranges (SIRS: 38.6 ± 1.16°C and non‐SIRS: 39.2 ± 0.7°C; *P* < .003). The proportion of calves presenting in standing or in lateral recumbency was significantly different between the SIRS group (40% standing and 55% in lateral recumbency) compared with the non‐SIRS group (74% standing and 23% in lateral recumbency; *P* < .001; Table S2).

The serum Hp concentrations were similar between SIRS (0.29 g/L; range, 0.05‐3.6) and non‐SIRS calves (0.22 g/L; range, 0‐4.2; *P* = .62; Table S3). Calves with SIRS had significantly higher serum globulin concentrations (25 g/L; range, 10‐46) than the non‐SIRS group (22 g/L; range, 9‐59; *P* = .05). The albumin/globulin ratio was significantly lower in calves with SIRS (1.05; range, 0.4‐2.7) than the non‐SIRS group (1.3; range, 0.5‐2.4; *P* < .007). L‐lactate was significantly higher in calves with SIRS (3.1 mmol/L; range, 0.5‐22) than non‐SIRS group (2 mmol/L; range, 0‐14; *P* < .01). GLDH activity was similar between SIRS (12 U/L; range, 1‐1025) and non‐SIRS calves (9 U/L; range, 2‐137; *P* = .2). The AST activity was also similar between the SIRS (55 U/L; range, 24‐19 140) and non‐SIRS calves (181 U/L; range, 50‐24 875; *P* = .31). The GGT activity was significantly higher in SIRS (124 U/L; range, 8‐1576) than non‐SIRS calves (60 U/L; range, 12‐952; *P* = .03; Table S4).

### 3.2 Survivors and nonsurvivors

A total of 22 (22%) calves died (2/22; 9%) or were euthanized (20/22; 81%) during hospitalization. Postmortem examination diagnosis of nonsurviving calves included sepsis (n = 14), peritonitis (n = 3), ischemic strangulating obstruction of the small intestine (n = 1), prematurity and omphalitis (n = 1), flexural deformities (n = 1), cardiac congenital abnormalities (n = 1), and liver failure of unknown origin (n = 1). Hospitalization time was longer in surviving (7 days; range, 1‐25) than nonsurviving calves (2 days; range, 1‐26; *P* < .001; Table 1). Age, posture at presentation, suckling reflex quality, the presence of diarrhea, and pneumonia were significantly different between surviving and nonsurviving calves (Table 1). The number of calves classified as having SIRS were significantly higher in the nonsurviving (81%) than in the surviving group (51%; *P* = .02). The serum Hp concentration was similar between survivors and nonsurvivors (Table 2). The absolute WBC, neutrophil count, and total serum protein, albumin and globulin concentrations were significantly lower in nonsurvivors than survivors (*P* < .05, for all comparisons). The absolute number of bands was significantly higher in nonsurvivor than in survivor calves (*P* < .001). Serum creatinine, sodium, and whole blood lactate concentrations and GLDH and AST activities were significantly higher in nonsurviving than surviving calves (*P* < .05, for all comparisons). GGT activity was similar between surviving (75 U/L; range, 12‐952) and nonsurviving calves (125 U/L, 8‐1576; *P* = .73; Table 2).

**TABLE 1:** Physical examination findings obtained on admission and hospitalization times of 102 survivors and nonsurvivors sick calves <30 days of age

Variable	Survivors (n = 80)	Nonsurvivors (n = 22)	*P*‐value
Age (days)	14 (1–30)	3 (1‐30)	.003
Sex			.22
Male	32 (40%)	10 (45%)	
Female	48 (60%)	12 (56%)	
Breed			.87
Holstein	70 (88%)	20 (90%)	
Jersey	6 (7%)	1 (5%)	
Other	4 (5%)	1 (5%)	
Attitude			.34
Bright	7 (9%)	4 (5%)	
Obtunded	55 (70%)	13 (59%)	
Comatose	17 (21%)	8 (36%)	
Posture			.001
Standing	57 (72%)	7 (32%)	
Sternal	2 (3%)	0	
Lateral	20 (25%)	15 (68%)	
Suckling reflex			.001
Strong	50 (63%)	5 (23%)	
Weak	23 (29%)	8 (36%)	
Absent	6 (8%)	9 (41%)	
Heart rate (bpm)	120 (52‐210)	147 (68‐190)	.08
Respiratory rate (rpm)	36 (12‐100)	56 (24‐140)	.001
Temperature (°C)	39 (35.1‐40.8)	39 (36.5‐41.6)	.66
Diarrhea	67 (85%)	9 (41%)	.001
Pneumonia	19 (25%)	12 (55%)	.007
SIRS^a^	32 (51%)	17 (81%)	.02
Dehydration			.07
None	16 (20%)	3 (14%)	.67
Mild (5%‐6%)	32 (40%)	7 (32%)	
Moderate (7%‐9%)	22 (28%)	8 (36%)	
Severe (>10%)	10 (12%)	4 (18%)	
Days in hospital	7 (1–25)	2 (1–26)	.001

*Note*: References ranges for heart rate (100‐140 bpm); respiratory rate (30‐60 rpm); temperature (38.5‐39.4°C). *P*‐values obtained from *t*‐student or Mann‐Whitney *U*‐tests, while *P*‐values for categorical were obtained from Fisher exact or *X* ^2^ tests.

Abbreviations: bpm, beats per minute; rpm, respirations per minute; SIRS, systemic inflammatory response syndrome.

^a^ Data for SIRS classification was only available for 84 calves.

**TABLE 2:** Hematologic and serum biochemistry variables obtained on admission from 102 surviving and nonsurviving sick calves <30 days of age

Variable	Survivors	Nonsurvivors	Reference range^b^ ^,^ ^18^, ^19^, ^20^	*P*‐value
RBC^a^ (× 10^12^/L)	7 (3.8‐13.4)	6.3 (3.9‐9.3)	4.9‐7.5	.05
WBC^a^ (× 10^9^/L)	9.8 (3.4‐32)	5.7 (1.2‐18)	5‐13	.01
Neutrophils^a^ (× 10^9^/L)	5.1 (1.1‐26)	1.5 (0.03‐12)	1.7‐6	.001
Bands^a^ (× 10^9^/L)	0.1 (0‐6.1)	0.4 (0‐2.1)	0‐0.2	.04
Lymphocytes^a^ (× 10^9^/L)	2.5 (0.2‐7)	1.9 (0.7‐6)	1.8‐8.1	.05
Monocytes^a^ (× 10^9^/L)	0.6 (0‐2.8)	0.4 (0‐2.2)	0.1‐0.7	.19
Haptoglobin (g/L)	0.23 (0–4.2)	0.2 (0.05‐0.9)	0‐0.5	.48
Total serum protein (g/L)	56 ± 12	47 ± 10	66‐86	.001
Albumin (g/L)	29 ± 6.2	24 ± 6.5	30‐42	.006
Globulin (g/L)	25 (9‐59)	23 (10‐35)	30‐53	.03
Albumin/globulin ratio	1 (0.45‐2.4)	1 (0.4‐2.7)	N/A	.97
BHBA (μmol/L)	83 (0‐534)	34 (0‐375)	324‐1296	.02
Glucose (mmol/L)	4.8 (1.5‐18)	4.9 (0.1‐12)	2.6‐4.4	.34
Urea (mmol/L)	5.6 (1.3‐103)	4.7 (1.8‐31)	3‐8	.78
Creatinine (μmol/L)	92 (7.1‐561)	152 (61‐1025)	34‐88	.01
GLDH (U/L)	9 (1‐137)	20 (5‐1280)	3‐52	.001
GGT (U/L)	75 (12‐952)	125 (8‐1576)	11‐51	.73
AST (U/L)	48 (1‐3107)	65 (24‐4062)	44‐153	.02
CK (U/L)	193 (27‐24 875)	198 (37‐19 140)	44‐211	.94
pH	7.3 (6.7‐7.5)	7.3 (6.9‐7.46)	7.31‐7.53	.67
PvCO_2_ (mm Hg)	50 ± 12	55 ± 13	35‐44	.13
HCO_3_^−^ (mmol/L)	26 (4.6‐53)	26 (5.4‐38.1)	17‐29	.6
BE (mmol/L)	1.4 (−26 to 28)	2.2 (−23 to 15)	−3.5 to 3.5	.71
Na^+^ (mmol/L)	136 (113‐147)	140 (118‐154)	132‐145	.005
K^+^ (mmol/L)	4.5 (2‐11)	4.9 (3.7‐6.3)	3.9‐5.6	.33
Cl^−^ (mmol/L)	96 (69‐123)	97 (67‐111)	90‐113	.85
iCa^++^ (mmol/L)	1.3 (0.8‐2.5)	1.3 (0.9‐1.7)	1.2‐1.5	.2
L‐Lactate (mmol/L)	2.5 (0‐14)	6.2 (0.4‐22)	0‐2	.001
AG (mmol/L)	17 (3.8‐38)	21 (6.4‐36)	13‐22	.07
TS (g/L)	61 ± 11	55 ± 10	60‐80	.01

*Note*: *P*‐values obtained from *t*‐student or Mann‐Whitney *U*‐tests, while *P*‐values for categorical were obtained from Fisher exact or *X* ^2^ tests.

Abbreviations: AG, anion gap; AST, aspartate aminotransferase; BE, base‐excess; BHBA, ß‐hydroxybutyrate; CK, creatine kinase; GGT, gamma‐glutamyl transferase; GLDH, glutamate dehydrogenase; HCO_3_^−^, bicarbonate; iCa^2+^, ionized calcium; PvCO_2_, partial venous pressure of carbon dioxide; RBC, red blood cells count; TS, total solids; WBC, white blood cells count.

^a^ Values available only for 84 calves.

^b^ Reference values provided by the institutional animal health laboratory based on samples obtained from 60 clinical healthy cows from 10 different farms.

### 3.3 Clinicopathologic variables associated with survival of sick calves

Age, sex, posture, suckling reflex, HR, RR, mucus membrane color, WBC count, neutrophil, lymphocyte and monocyte count, along with the concentrations of total protein, albumin, globulin, creatinine, glucose, GGT, AST, and GLDH were significant in univariable analysis and offered to the multivariable model (Table S1). In the final model, suckle reflex quality, HR, neutrophil count, and GGT concentration were significant (Table 3). Specifically, calves with a strong suckle reflex and a normal HR had a lower hazard of nonsurvival. Calves with a neutrophil count below the reference range had a higher hazard of nonsurvival and as the concentrations of GGT rose, the hazard of mortality increased. The Harrell's C concordance statistic was 0.93, indicating the model could correctly order survival times for pairs of calves 93% of the time based on HR, quality of suckling reflex, neutrophil count, and GGT concentration.

**TABLE 3:** Results of the cox proportional hazards model evaluating the association between clinicopathological variables on admission and survival of 84 sick, hospitalized calves

Variables		Haz. ratio	SE	*P*	95% CI
Suckling reflex	Strong				
	Weak	4.09	3.17	.07	0.89‐18.71
	Absent	6.44	4.93	.02	1.44‐28.86
Heart rate	Normal				
	<100 bpm	12.2	9.77	.002	2.54‐58.62
	>140 bpm	3.59	2.26	.04	1.05‐12.33
Neutrophil count	Normal				
	<1.7 × 10^9^/L	7.36	4.83	.002	2.03‐26.66
	>6 × 10^9^/L	0.38	0.31	.24	0.08‐1.89
GGT	Every 50‐unit increase	1.12	0.00	.008	1.03‐1.21

Abbreviations: bpm, beats per minute; CI, confidence interval; GGT, gamma‐glutamyl transferase; haz. ratio, hazard ratio.

## 4 DISCUSSION

In the present study, no significant differences were identified in Hp concentration of calves with or without SIRS. Similarly, admission serum Hp concentration was not associated with survival of hospitalized sick calves. This is in contrast to our hypothesized outcomes, in which we expected associations between serum Hp with severity of the disease (eg, SIRS) or survival in sick calves. We based our hypotheses on previous studies in calves showing that serum or blood concentration of Hp in diarrheic calves,^6^ and calves with respiratory disease complex^7^, ^8^ correlated with the severity of clinical signs. Higher Hp concentration occurs in calves with fever, obtundation and severe dehydration compared to calves without mild clinical signs.^6^ Additionally, in humans with sepsis^5^ and horses with colic,^21^ plasma Hp are higher in survivors than nonsurvivors. The reasons for the lack of association between serum Hp concentration and severity of illness or survival in our study are unknown. However, similar to our results, no association between Hp concentrations at hospital admission and survival was identified in cats,^22^ critically ill foals^23^ or humans with sepsis.^24^ Our results indicated that in the study sample of calves included in this study, Hp was useful to identify an ongoing inflammatory process; however, its ability to predict severity of disease or survival was poor. Future studies should investigate whether longitudinal monitoring, rather than a single Hp measurement, can better predict survival of sick calves as reported with other acute phase proteins in dogs.^25^

The GLDH activity, an indicator of hepatic insult, was not a predictor of survival in the calves included in our study based on multivariable analysis; however, GLDH activity was higher in nonsurviving than surviving calves. Similarly, serum activity of liver‐derived enzyme (GGT and SDH) is increased in foals with sepsis, but they are not useful predictors of survival.^15^ Primary hepatic disease was only reported in 1 calf included in this study, and high GLDH activity was most commonly identified secondary to other diseases, such as sepsis, diarrhea, and pneumonia. Therefore, high GLDH activity likely resulted from hepatic insult associated with hypoperfusion, tissue hypoxia or hematogenous dissemination of bacteria or endotoxin.^26^, ^27^

The number of calves with SIRS was higher in the nonsurviving than in the surviving group. In addition, the clinical diagnoses and postmortem examinations of nonsurviving calves indicated that most of the calves died because of sepsis (n = 17/22). This could explain the association of higher percentages of dehydration, L‐lactate, and creatinine concentration in nonsurviving calves in the univariable analysis, and the association of low neutrophil count with nonsurvival identified in the multivariable analysis. High GGT activity was also associated with nonsurvival. In contrast, low serum GGT activity (<31 U/L) was reported as a risk factor for nonsurvival of sick neonatal calves with diarrhea.^16^ The authors of that study proposed that lack of consumption of colostrum, suggested by the low GGT activity, and therefore failure of transfer of passive immunity (FTPI) could explain the association between low GGT activity and mortality of sick neonatal calves with diarrhea in an age‐dependent manner. In our study, it is likely that nonsurviving calves consumed colostrum, as suggested by the high serum GGT activity, but nothing is known about the IgG concentration in the consumed colostrum because the predictor capacity of GGT activity for transfer of passive immunity is poor.^28^ No information was available regarding TPI in our calves, thus it is possible that FTPI could still have played a role in predisposing calves to sepsis.

A potential source of serum GGT activity is an insult to the biliary epithelium. Primary hepatic disease was only documented in a single calf; however, structural cholestasis, biliary necrosis and cholestasis secondary to endotoxin‐induced inhibition of bile salt uptake could have contributed to the increase in serum GGT activity.^29^, ^30^ Age might also be a contributing factor to the changes in liver enzyme activity.^18^, ^31^ The retrospective nature of our study prevented us from determining the specific source of GGT activity elevation in this group of calves.

Limitations of this study include its retrospective nature, the subsequent lack of standardized data collection, and the lack of categorization of clinical signs and treatments, which prevented assessment of the effect of individual treatment variations on the survival analysis. Economic constraints could have contributed to the decision to euthanize calves in the nonsurvival group. However, the diagnosis of most euthanized calves had a grave prognosis regardless of the available economic resources. Our study sample was a heterogeneous group of sick calves with different levels of sickness. Thus, we classified the calves as SIRS and non‐SIRS, and our results are comparable to those reported previously in hospitalized calves with diarrhea using the same SIRS classification criteria.^16^ An additional limitation of our study was that the reference ranges used to dichotomize selected variables of the biochemical profile were based on values for calves 3 to 4 weeks of age or for adult cattle. The rational for choosing these references ranges was that in our study 70% (n = 72) calves were > 7 days of age (mean age 12 days) and biochemical variables of calves >1 week of age are comparable to those obtained from adult animals.^18^, ^31^ Similarly, reference ranges for hematological variables of calves >5 days of age are comparable to those from 3 to 4 weeks of age.^19^, ^20^, ^32^ Another limitation of our study is that only 38% of potentially eligible sick calves admitted during the study period fulfilled the inclusion criteria which would likely tend to bias the study toward sicker or high‐risk septic calves. Therefore, the results of our study should be interpreted taking this limitation into consideration when extrapolating to a different study sample.

In summary, serum Hp concentrations and GLDH activity were not useful in predicting SIRS or nonsurvival in sick, hospitalized calves younger than 30 days of age enrolled in this study. In contrast, suckle reflex quality, HR, neutrophil count, and GGT concentration were predictive of mortality.

## CONFLICT OF INTEREST DECLARATION

Authors declare no conflict of interest.

## OFF‐LABEL ANTIMICROBIAL DECLARATION

Authors declare no off‐label use of antimicrobials.

## INSTITUTIONAL ANIMAL CARE AND USE COMMITTEE (IACUC) OR OTHER APPROVAL DECLARATION

Authors declare no IACUC or other approval was needed.

## HUMAN ETHICS APPROVAL DECLARATION

Authors declare human ethics approval was not needed for this study.

## Supporting information


**Table S1** Results of the cox proportional hazards model (univariable) evaluating the association between clinicopathological variables on admission and survival of sick, hospitalized calves.

**Table S2** Physical examination and hospitalization time results of 84 calves <30 days hospitalized for different clinical condition and classified as SIRS and Non‐SIRS.

**Table S3** Admission complete blood cell count and selected serum biochemistry variables values of 84 hospitalized sick calves <30 days of age with and without SIRS.

**Table S4** Serum biochemistry variables obtained on admission from 84 hospitalized calves <30 days of age with or without SIRS.
