# Supplementary material for: Serum haptoglobin concentration and liver enzyme activity as indicators of systemic inflammatory response syndrome and survival of sick calves
Source: J Vet Intern Med. 2022 Jan 18;36(2):812–9. doi: 10.1111/jvim.16357 (PMC8965222; doi:10.1111/jvim.16357)
Supplement: Supplementary file 1 — Table S1 Results of the cox proportional hazards model (univariable) evaluating the association between clinicopathological variables on admission and survival of sick, hospitalized calves. [file JVIM-36-812-s004.pdf]

**Supplementary Table 1.** Results of the cox proportional hazards model (univariable) evaluating the association between clinicopathological variables on admission and survival of sick, hospitalized calves.					
**Variables**		**Haz. Ratio**	**Std. Err.**	**P-value**	**95% Conf. Int**
Age	Every 1-unit increase	0.92	0.02	.013	0.87 to 0.98
Breed	Holstein Friesian				
	Jersey	0.73	0.75	.767	0.09 to 5.52
	Other	0.99	1.01	.99	0.13 to 7.43
Sex	Female				
	Male	1.76	0.77	.20	0.74 to 4.18
Attitude	Bright				
	Obdunded	1.39	1.45	.74	0.18 to 10.7
	Comatose	1.99	2.14	.52	0.24 to 16
Posture	Standing				
	Sternal	5.34	2.59	.001	2.05 to 13.8
Suckling Reflex	Strong				
	Weak	4.09	3.17	.07	0.89 to 18.7
	Absent	6.44	4.93	.02	1.44 to 28.8
Heart Rate	Normal				
	< 100 bpm	12.20	9.77	.002	2.54 to 58.6
	> 140 bpm	3.59	2.26	.04	1.05 to 12.3
Respiratory Rate	Normal				
	1	0.13	0.13	.05	0.01 to 0.99
	2	1.10	0.62	.85	0.36 to 3.32
Temperature	Every 1-unit increase	1.02	0.19	.89	0.70 to 1.49
Mucus membranes	Normal				
	Congestive	1.12	0.89	.88	0.23 to 5.35
	Pale	5.80	2.76	0	2.28 to 14.7
Dehydration	None to 5%				
	6 to 8%	0.76	0.46	.65	0.22 to 2.53
	> 8%	1.37	0.66	.51	0.52 to 3.56
Diarrhea	No				
	Yes	0.21	0.09	.001	0.09 to 0.51
Pneumonia	No				
	Yes	2.81	1.23	.018	1.19 to 6.64
Red blood cells	Every 1-unit increase	0.78	0.11	.10	0.58 to 1.05
Total WBC	Normal				
	< 5 x 10^9/L	2.97	1.50	.03	1.10 to 8.02
	> 13.4 x 10^9/L	0.95	0.60	.94	0.27 to 3.29

Neutrophil count	Normal				
	< 1.7 x 10^9/L	7.36	4.83	.002	2.03 to 26.6
	> 6 x 10^9/L	0.38	0.31	.24	0.08 to 1.89
Bands	Normal				
	> 0.22 x 10^9/L	1.88	0.85	.16	0.77 to 4.56
Lymphocytes	Every 1-unit increase	0.61	0.12	.01	0.41 to 0.91
Monocytes	Every 1-unit increase	0.41	0.18	.04	0.17 to 0.97
Basophiles	Normal				
	> 0.08 x 10^9/L	1.35E-14	1.70E-07	1	0 to
Eosinophiles	Normal				
	> 0.05 x 10^9/L	0.96	0.99	.97	0.12 to 7.26
Neutrophil toxicity	None				
	mild	2.90	2.25	.16	0.63 to 13.2
	Moderate	5.36	3.62	.01	1.42 to 20.1
	Severe	13.4	8.37	0	3.98 to 45.5
Total protein	Every 1-unit increase	0.93	0.01	.002	0.89 to 0.97
Albumin	Every 1-unit increase	0.91	0.03	.01	0.85 to 0.98
Globulin	Every 1-unit increase	0.91	0.02	.007	0.86 to 0.97
Alb/glob ratio	Every 1-unit increase	1.51	0.69	.36	0.61 to 3.70
Urea	Normal				
	> 8.6 mmol/L	0.73	0.37	.55	0.26 to 2.02
Creatinine	Every 1-unit increase	1.00	0.0009	.01	1.00 to 1.00
Glucose	Normal				
	< 2.6 mmol/L	5.67	4.34	.02	1.26 to 25.4
	> 4.4 mmol/L	2.16	1.37	.22	0.61 to 7.54
GGT	Every 1-unit increase	1.002	0.00	.008	1.001 to 1.004
AST	Normal				
	> 155 U/L	3.05	1.70	.04	1.02 to 9.13
CK	Normal				
	> 214 U/L	1.22	0.53	.63	0.51 to 2.90
GLDH	Normal				
	> 52 U/L	2.95	1.45	.02	1.12 to 7.76
BHBA	Every 1-unit increase	0.99	0.002	.59	0.99 to 1.00
Haptoglobin	Every 1-unit increase	0.35	0.29	.21	0.06 to 1.83
Haz. Ratio, hazard ratio; Conf. Int, confidence interval; bpm, beats per minute; GGT: Gamma-Glutamyl transferase.					
