# Supplementary material for: Serum haptoglobin concentration and liver enzyme activity as indicators of systemic inflammatory response syndrome and survival of sick calves
Source: J Vet Intern Med. 2022 Jan 18;36(2):812–9. doi: 10.1111/jvim.16357 (PMC8965222; doi:10.1111/jvim.16357)
Supplement: Supplementary file 2 — Table S2 Physical examination and hospitalization time results of 84 calves <30 days hospitalized for different clinical condition and classified as SIRS and Non‐SIRS. [file JVIM-36-812-s003.pdf]

Supplementary Table 2: Physical examination and hospitalization time results of 84 calves < 30 days hospitalized for different clinical condition and classified as SIRS and Non-SIRS.

Variable	SIRS n= 49	Non-SIRS n= 35	P - Value
Age [days]	8 [1 - 30]	13 [1 - 30]	.074
Sex			.852
Male	22 [45%]	15 [43%]	
Female	27 [55%]	20 [57%]	
Breed			.937
Holstein	43 [88%]	30 [86%]	
Jersey	4 [8%]	3 [8%]	
Other	2 [4%]	2 [6%]	
Attitude			.16
Bright	3 [6%]	5 [14%]	
Obtunded	30 [61%]	24 [69%]	
Comatose	16 [33%]	6 [17%]	
Position			.008
Standing	20 [40%]	26 [74%]	
Sternal	2 [4%]	1 [3%]	
Lateral	27 [55%]	8 [23%]	
Suckling reflex			.06
Strong	17 [35%]	20 [57%]	
Weak	20 [40%]	12 [34%]	
Absent	12 [25%]	3 [9%]	
Diarrhea	33 [67%]	28 [80%]	.17
Pneumonia	20 [40%]	10 [29%]	.29
Heart rate [bpm]	130 ± 36	124 ± 12	.28
Respiratory rate [rpm]	44 [12 - 140]	40 [24 - 80]	.91
Temperature [˚C]	38.6 ± 1.16	39.2 ± 0.7	.003
Dehydration			.112
None	6 [12%]	11 [31%]	
Mild [5 – 6 %]	16 [33%]	9 [26%]	
Moderate [7 – 9%]	17 [35%]	12 [34%]	
Severe [> 10%]	10 [20%]	3 [8%]	
Days in hospital	5 [1 - 26]	5 [1 - 24]	.76

bpm, beats per minute; rpm, respirations per minute; SIRS, systemic inflammatory response syndrome. References ranges for heart rate [100 to 140 bpm]; respiratory rate [30 to 60 rpm]; temperature [38.5 to 40.5 ˚Celsius]. SIRS, systemic inflammatory response syndrome. P-values obtained from *t*-student or Mann-Whitney U-tests, while P-values for categorical were obtained from Fisher exact or X^2 tests.
