# Supplementary material for: Serum haptoglobin concentration and liver enzyme activity as indicators of systemic inflammatory response syndrome and survival of sick calves
Source: J Vet Intern Med. 2022 Jan 18;36(2):812–9. doi: 10.1111/jvim.16357 (PMC8965222; doi:10.1111/jvim.16357)
Supplement: Supplementary file 3 — Table S3 Admission complete blood cell count and selected serum biochemistry variables values of 84 hospitalized sick calves <30 days of age with and without SIRS. [file JVIM-36-812-s002.pdf]

Supplementary Table 3. Admission complete blood cell count and selected serum biochemistry variables values of 84 hospitalized sick calves < 30 days of age with and without SIRS.

Variable	SIRS	Non-SIRS	Ref. Range	P - Value
RBC [x 10^{12}/L]	6.8 ± 1.6	7.52 ± 2.1	4.9 – 7.5	.11
WBC [x 10^{9}/L]	8.8 [1.2 – 32]	8.8 [2.8 – 30]	5 – 13	.89
Neutrophils [x 10^{9}/L]	4.8 [0.03 – 26]	4 [0.5 – 22]	1.7 – 6.0	.53
Bands [x 10^{9}/L]	0.1 [0 – 6.1]	0.1 [0 – 3.2]	0 – 0.2	.85
Lymphocytes [x 10^{9}/L]	2.3 [0.75 – 5.8]	2.5 [0.2 – 7]	1.8 – 8.1	.39
Monocytes [x 10^{9}/L]	0.5 [0 – 2.8]	0.6 [0 – 2.7]	0.1 – 0.7	.78
Haptoglobin [g/L]	0.22 [0 – 4.2]	0.29 [0.05 – 3.6]	0 – 0.5	.62
Total protein [g/L]	50 [23 – 79]	51 [28 – 86]	66 – 86	.73
Albumin [g/L]	26 ± 6.2	28 ± 4.8	30 – 42	.21
Globulin [g/L]	25 [10 – 46]	22 [9 – 59]	30 – 53	.05
Albumin/globulin ratio	1.05 [0.4 – 2.7]	1.3 [0.5 – 2.4]		.007

SIRS, systemic inflammatory response syndrome; RBC: Red blood cells, WBC: White blood cells. P-values obtained from *t*-student or Mann-Whitney U-tests, while P-values for categorical were obtained from Fisher exact or X^2 tests.
