# Supplementary material for: Serum haptoglobin concentration and liver enzyme activity as indicators of systemic inflammatory response syndrome and survival of sick calves
Source: J Vet Intern Med. 2022 Jan 18;36(2):812–9. doi: 10.1111/jvim.16357 (PMC8965222; doi:10.1111/jvim.16357)
Supplement: Supplementary file 4 — Table S4 Serum biochemistry variables obtained on admission from 84 hospitalized calves <30 days of age with or without SIRS. [file JVIM-36-812-s001.pdf]

**Supplementary Table 4.** Serum biochemistry variables obtained on admission from 84 hospitalized calves < 30 days of age with or without SIRS.				
**Variable**	**SIRS**	**No SIRS**	**Ref. Range**	**P - Value**
BHBA [µmol/L]	61 [0 - 534]	65 [0 - 294]	324 – 1296	.74
Glucose [mmol/L]	5 [0.1 - 162]	5 [1.1 – 10]	2.5 – 4.3	.98
Urea [mmol/L]	5 [1.3 – 46]	4.8 [1.3 - 103]	3.0 – 8.0	.57
Creatinine [µmol/L]	106 [43 - 1025]	93 [7.1 - 559]	34 - 88	.50
GLDH [U/L]	12 [1 - 1280]	9 [2 - 137]	3 – 45	.20
GGT [U/L]	124 [8 - 1576]	60 [12 - 952]	11 - 51	.03
AST [U/L]	55 [24 - 4062]	48 [1 - 3107]	44 - 153	.31
CK [U/L]	169 [27 – 19,140]	181 [50 – 24,875]	44 – 211	.99
pH	7.3 [6.9 – 7.5]	7.34 [6.99 – 7.47]	7.31 – 7.53	.14
$PvCO_2$ [mmHg]	55 ± 14	48 ± 11	35 -44	.01
HCO_3^- [mmol/L]	26 ± 10	24 ± 8.5	17 – 29	.72
BE [mmol/L]	2.6 [-23 – 28]	1.5 [-21 to 12]	-3.5 to 3.5	.59
Na^+ [mmol/L]	138 [113 - 154]	136 [116 - 147]	132 – 145	.32
K^+ [mmol/L]	4.5 [2.7 – 11]	4.5 [2 – 9]	3.9 – 5.6	.67
Cl^- [mmol/L]	96 [67 - 113]	96 [83 - 119]	90 – 113	.36
iCa^{++} [mmol/L]	1.3 [.8 – 2.53]	1.3 [1.1 – 1.7]	1.2 – 1.5	.40
L-Lactate [mmol/L]	3.1 [0.5 - 22]	2 [0 – 14]	0 - 2.0	.01
AG [mmol/L]	15 [11 – 38]	18 [3.8 – 37]	13 – 22	.05
TS [g/L]	60 ± 11	59 ± 11	60 – 80	.59

BHBA: ß-Hydroxybutyrate, GLDH: Glutamate dehydrogenase, GGT: Gamma-Glutamyl transferase, AST: Aspartate Aminotransferase, CK: Creatine kinase, $PvCO_2$: Partial venous pressure of carbon dioxide, HCO_3^-: Bicarbonate, BE: Base-excess, iCa^{++}: Ionized calcium, AG: Anion gap, TS: Total solids. P-values obtained from *t*-student or Mann-Whitney U-tests, while P-values for categorical were obtained from Fisher exact or X^2 tests.
